# Daphnia as an Emerging Epigenetic Model Organism

**DOI:** 10.1155/2012/147892

**Published:** 2012-01-29

**Authors:** Kami D. M. Harris, Nicholas J. Bartlett, Vett K. Lloyd

**Affiliations:** Department of Biology, Mount Allison University, 63B York Street, Sackville, NB, Canada E4L 1G7

## Abstract

*Daphnia* offer a variety of benefits for the study of epigenetics. *Daphnia's* parthenogenetic life cycle allows the study of epigenetic effects in the absence of confounding genetic differences. Sex determination and sexual reproduction are epigenetically determined as are several other well-studied alternate phenotypes that arise in response to environmental stressors. Additionally, there is a large body of ecological literature available, recently complemented by the genome sequence of one species and transgenic technology. DNA methylation has been shown to be altered in response to toxicants and heavy metals, although investigation of other epigenetic mechanisms is only beginning. More thorough studies on DNA methylation as well as investigation of histone modifications and RNAi in sex determination and predator-induced defenses using this ecologically and evolutionarily important organism will contribute to our understanding of epigenetics.

## 1. Introduction

The unusual life cycle of the freshwater microcrustacean, *Daphnia*, has been studied for more than 150 years [1]. Most species are cyclic parthenogens able to produce two types of eggs, diploid parthenogenetic eggs or haploid sexual eggs, in response to environmental cues [2, 3]. Sex determination is likewise environmentally controlled; males are produced in response to suitable environmental cues [3]. Additionally, *Daphnia* exhibit a range of spectacular polyphenisms, phenotypic alternations including helmet and neckteeth formation, in response to predators [4, 5]. This makes *Daphnia* an excellent candidate for studying environmental influences on epigenetic developmental programs. Most importantly in the context of epigenetics, clonal lines are genetically identical yet consist of phenotypically divergent individuals. This offers a unique opportunity to separate genetic and epigenetic influences on the phenotype, an invaluable asset when studying epigenetics. The attractiveness of *Daphnia* as a potential epigenetic model organism is further enhanced by the fact that they are easy and inexpensive to maintain and have a rapid life cycle. As a primary consumer and a food source for invertebrates and fish [6], there is an extensive body of literature on their ecological role, development, and the evolution of parthenogenesis. Thus, *Daphnia *is an ecologically important organism well-studied in the context of evolution, ecology, ecotoxicology, predator-induced polyphenisms, and genomics [7, 8] and offers unparalleled opportunities to study epigenetics in these biologically important processes.

Epigenetics is the study of mitotically or meiotically heritable changes in phenotypes that occur without changes in the DNA sequence [9]. Altered gene expression can be caused by DNA methylation, histone modifications, and RNA interference as well as other, less well-studied, epigenetic mechanisms such as variant histones, nucleosome phasing, higher-order chromatin structures, and nuclear localization [4, 9].

DNA methylation, performed by either *de novo* or maintenance DNA methyltransferases, has been associated with transcriptional regulation, chromosome inactivation, and transposable element regulation, among other functions [10]. Although DNA methylation is found in a wide variety of eukaryotes, the amount of methylation and its organization within the genome differ dramatically between species and developmental stages [4]. DNA methylation interacts with other epigenetic processes [11]. Modifications to the amino- or carboxyl-terminal histone tails affect the interactions of histones with DNA, other histones, and other chromatin-associated proteins [12]. These modifications are performed by specialized enzymes and include acetylation, ubiquitination, sumoylation, phosphorylation, and methylation, all of which can alter gene expression [12]. DNA methyltransferases and histone modifying enzymes can recruit each other by way of a mutual attraction to the modifications imposed by the other [11]. DNA methylation and histone modifications also interact with the RNA silencing system [13]. The RNA silencing system operates through the production of small noncoding RNA molecules (ncRNA) and is referred to as RNA interference (RNAi). Small RNAs, microRNA (miRNA) and short interfering RNA (siRNA) are excised from larger double-stranded molecules can form RNA-induced silencing complexes (RISC) that target complementary nucleic acid sequences and recruit or activate DNA methyltransferases and histone modifying enzymes [14].

Epigenetic marks are modified by external environmental factors such as nutrition and exposure to chemicals, as well as developmental cues [15]; these epigenetic alterations can enhance the cell and organism's ability to respond to its environment and thrive [16]. DNA methylation, histone modifications, and RNAi are all mitotically transmissible. Additionally, as epigenetic changes can be adaptive, selection for meiotic transmission might be expected to allow epigenetic information to be passed between generations [4]. Such transgenerational inheritance has been documented in* Arabidopsis* [17], mice [18], *Drosophila* [19], and humans [20, 21] and is postulated in *Daphnia* [16]. However, identification of transgenerational effects can be problematic when the embryo undergoes development in the mother's body, as is the case in *Daphnia*. In such situations, maternal exposure to environmental factors could affect the offspring either by retention of maternal epigenetic states in the germ line cells that give rise to the embryo, a true transgenerational effect, or more simply by exposure of the somatic cells of the embryo while it is in the mother. To resolve this ambiguity, the persistence of the trait needs to be monitored in the F3 and subsequent generations, those which were not exposed as either the embryos that produce the F1 or the embryonic germ line that produce the F2.

Spurred by the use of *Daphnia* as a subject of ecological and developmental research, numerous techniques have been developed that can equally enhance its use in epigenetic studies. Conventional cytological methods have been employed [22] and more recently these have been extended to include fluorescence *in situ* hybridization (FISH) [23]. This could allow examination of higher-order chromatin structures that have been associated with the epigenetic status of genome regions in other animals [24]. Recently *Daphnia pulex* was the first crustacean to have its genome sequenced, revealing the largest number of genes yet found in a single organism, yet present in a remarkably compact genome [25]. The large number of genes is due to a very high rate of tandem gene duplication events, and approximately 30% of the genes are unique to *Daphnia* [25]. The availability of the genome sequence allows for the development of microarrays for genome-wide transcriptional studies [26]. *Daphnia* embryos are transparent and can develop independently of the mother, and as a result embryogenesis of *Daphnia* has been well documented [2, 27, 28]. With the genomic sequence available, conventional embryology can be extended to look at specific gene products. Methods for* in situ *immunohybridization and immunohistology have been developed so the tissue- and developmental-specific localization of RNAs and proteins can be examined [29]. In the context of epigenetics, this approach could be used to detect developmental and tissue-specific histone modifications. While there are no immortalized cell lines currently available for *Daphnia*, methods for primary culture have been developed [30]. These cells are viable for at least one week and can be transformed to study the role of overexpression of endogenous or foreign genes [30]. More recently, Kato et al. [31, 32] showed that it is possible to insert double-stranded RNA to reduce the expression of genes by RNAi-based gene knockdowns. The same technique can be used to over-express selected genes [33]. Knockdown of specific genes encoding for DNA methyltransferases, histone modifying enzymes, and their interacting proteins would allow for an assessment of the role of DNA methylation, histone modification, and related epigenetic processes correlated with the well-defined phenotypes that arise from epigenetic alterations.

## 2. The *Daphnia* Life Cycle and Epigenetic Phenotypic Variation

### 2.1. The *Daphnia* Life Cycle

**Figure 1:**
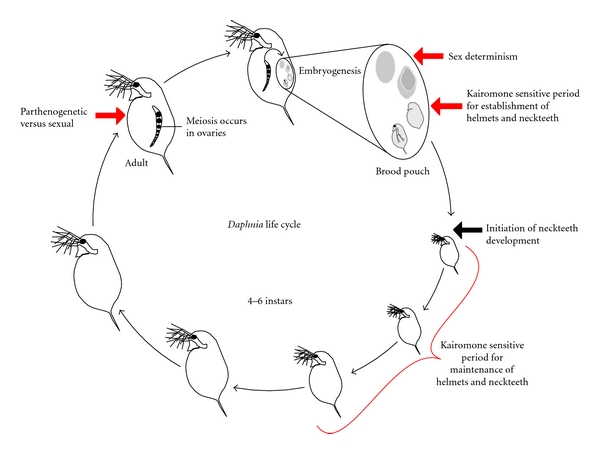
The *Daphnia* life cycle. The life cycle is shown with the stages at which the epigenome is sensitive to the environmental inputs that regulates sexual reproduction, sex determination, helmets, and neckteeth (indicated in red).

Most *Daphnia* can reproduce either asexually or sexually, depending on environmental cues. In both cases, eggs are produced by stem cells in the ovary [2]. In sexual eggs, meiosis is conventional and the haploid oocytes are fertilized. Parthenogenetic oocytes undergo only the equational meiotic division and so remain diploid and embryogenesis occurs without fertilization. Early embryogenesis commences as the egg matures on route to the brood pouch. Sexually produced eggs are typically produced in pairs, arrest in the blastula stage in the brood pouch, and the carapace overlying the brood pouch is modified into a tough, desiccation-resistant structure called the ephippium, which allows the eggs to survive harsh environmental conditions [2]. Parthenogenetic eggs complete embryogenesis in the brood pouch and are released as miniature versions of the adult [2]. Once hatched, the neonates typically undergo four to six larval instars, depending on species, before reaching reproductive maturity (Figure 1) [7, 38].

### 2.2. Epigenetic Regulation of the Life Cycle

Epigenetic changes in gene expression can modify an organism's phenotype and these changes are particularly obvious when there are no genetic differences between individuals of any one strain. Sensitivity of the epigenome to environmental cues occurs at different stages of the *Daphnia* life cycle. In general, the embryonic stages appear important for establishing the epigenetic states of genes involved in phenotypic variation, whereas exposure to environmental cues in the postembryonic larval stages is important for maintaining the epigenetic state (Figure 1).

The production of sexual versus asexual eggs is environmentally cued by environmental factors such as photoperiod, temperature, food abundance, and crowding [3]. In sexual eggs meiosis is conventional whereas asexual parthenogenetic eggs arise from an abortive first meiotic division, resulting in diploid eggs able to initiate development in the absence of fertilization [2]. In parthenogenetic eggs the first division is abortive; however, many of the same meiotic genes are expressed in parthenogenetic as in sexual reproduction [39] and bivalents are produced [40]. Nevertheless, genes suppressing recombination are overrepresented in the *Daphnia* genome relative to those promoting recombination [39], chiasmata are not observed [40] and genetic evidence of recombination has not been observed [41]. Thus, barring rare conversion, mitotic recombination or mutation events [42] parthenogenetic progeny are genetically identical. Since the ovary can simultaneously contain parthenogenetic and sexual eggs [2], the cues must act during the first meiotic division, as the oocytes form (Figure 1). How these environmental signals are interpreted and the molecular mechanism by which meiosis is regulated, remains unknown. The production of males is triggered by similar environmental cues as sexual egg production [3]; however, the control of male sex determination is independent of the regulation of female meiosis [2, 43]. Males are produced in either mixed or, more typically, all-male broods [3, 44, 45] and at least in some species can emerge from fertilized sexual ephippial eggs [46]. Despite obvious morphological differences—males being smaller, having testes, modified appendages, and carapace—all parthenogenetic offspring, male or female, and their mothers, are genetically identical. The mechanism of sex determination is thus clearly environmental and epigenetic. As juvenile hormone analogs induce males even in the absence of environmental cues, this suggests environmental cues are transduced by the endocrine system [33, 45]. Based on the production of intersexes in *D. magna* and *D. longispina*, induced by altered temperature or intermediate hormone concentrations, respectively, the determinative events in sex determination have been shown to act in oocyte maturation before the first embryonic division [44, 45]. Interestingly, Sanford [44] shows that intersex progeny are produced in broods long after the mother has been moved from the inducing conditions. This underscores the epigenetic nature of sex determination and might represent an example of transgenerational inheritance, but could equally reflect the early developmental action of the sex determination process. Many genes show differential expression between males and females [47], including the core sex-determination gene, *doublesex,* that is expressed at higher levels during embryogenesis in males than in females [33]. This suggests that, in *Daphnia,* environmental sex determination arose by imposing environmentally mediated regulation on the conserved *doublesex* genetic sex determination pathway. Identification of differences in the epigenetic status of the *doublesex* gene in males and females would further our understanding of environmental sex determination and the role of epigenetics in such a key aspect of the life cycle.

## 3. Epigenetic Regulation of Helmet Formation

Predators are an important aspect of an organism's environment, and various predator-induced defenses, such as helmets, have been well documented in *Daphnia* [16]. Helmets are cranial extensions of the exoskeleton that have been shown to decrease the daphnids' chances of predation [48]. Helmet growth is induced by kairomones, which are aquatic chemicals released by predators [48]. Circulating kairomones can double the relative helmet size in some daphnids [49].

Agrawal et al. [16] have shown that kairomones induce helmet growth in *Daphnia cucullata* both in the generation exposed to the kairomones and in their nonexposed progeny (Table 1). Newly hatched animals were exposed to kairomone-containing water, or control non-kairomone water and the size of helmets were monitored. Additionally, females were exposed and successive broods of their progeny were monitored for helmet production to detect transgenerational effects.

Exposure of neonates to kairomones induced helmet formation and removal of kairomones reduced helmet size [16]. This shows that kairomones act directly during early larval stages to promote helmet growth. Interestingly, when mothers were exposed, helmets were present in their neonate progeny, even if the progeny were not exposed [16]. Helmet formation in the neonates following only maternal exposure, could arise either from a transgenerational effect, transmission of the altered maternal epigenome to the F1 progeny via the oocyte, or, as the embryos are brooded in the mother, sensitivity of the embryonic somatic cells to kairomones. The latter is suggested by the fact that final helmet size is diminished in successive broods, which would have been younger, with fewer somatic cells, at the time of exposure, and that the F2 was not strongly influenced by grand-maternal exposure [16]. This finding also implies that late embryonic stages are more sensitive than earlier ones.

The effect of kairomone exposure on helmet size was cumulative; the largest helmets were obtained when both the mother and the neonates were exposed [16]. This additive effect indicates that both stages are sensitive. The possibility of different epigenetic events contributing to cuticular growth during embryonic and larval stages is suggested by similar studies on neckteeth formation (see below) [48]. Growth of the helmet is accomplished by mitotic division of diploid epidermal cells, thought to be triggered by signals from adjacent polyploid epidermal cells [50]. It is possible that kairomone exposure during late embryonic stages induces cell fate changes producing more polyploid cells whereas kairomone exposure during the larval stages increases the mitogenic activity of these polyploid cells.

## 4. Epigenetic Regulation of Neckteeth Formation

Another common predator-induced defense is exhibited by several species, including *Daphnia pulex. *In the presence of kairomones produced by *Chaoborus* (phantom midge) larvae,* Daphnia pulex* produces structures known as neckteeth, small protrusions on the neck region accompanied by a strengthened carapace [48, 51]. Daphnids that have these outgrowths have a higher predator escape rate, presumably due to the thickened exoskeleton that makes handling and consumption more difficult [48]. Development of the neckteeth begins in the first larval instar and growth continues until the third instar [52]. Withdrawal of the predatory cue at the first, second, or third instar reduces the number of neckteeth at successive instars [52]. Thus, the maintenance of epigenetic marks on the genes controlling the growth of neckteeth requires kairomone exposure in the larval stages [52]. However, Miyakawa et al. [51] were able to show that production of neckteeth involves at least two additional critical stages in late embryonic development. Few or no neckteeth form when kairomones are absent during embryogenesis, even if kairomones are present during the postembryonic larval stages [50]. Thus, as for helmet formation, embryonic exposure appears to be required to establish cell fates, while larval exposure is required to maintain and express the phenotype. *Differential Display 1* (*DD1*) is a gene identified as having altered expression in the embryonic stage in kairomone-exposed daphnids [51]. It is proposed that *DD1* plays a role in kairomone reception and/or cell fate determination that establishes the epigenetic state of target genes leading to the formation of neckteeth [51]. Multiple endocrine and morphogenetic genes, such as *Hox3, exd, JHAMT, Met, InR, IRS-1, DD1, DD2, *and* DD3,* were shown to be upregulated in the exposed postembryonic larvae [51]. The *Hox* gene upregulated in kairomone-exposed daphnids encodes a transcription factor associated with chromatin [53]. The *exd *and *met* gene products can similarly act as transcription factors and potentially alter the epigenetic status of downstream genes [54, 55]. Thus, the upregulation of these genes supports the conclusion that the maintenance and growth of neckteeth production is a result of epigenetic changes.

## 5. Epigenetic Regulation of Growth

In much the same way that external environmental cues such as kairomones can affect the development of helmets and neckteeth, environmental toxicants can affect the body length and growth in *Daphnia magna* [34]. Again, as the animals are all genetically identical, differences between exposed and nonexposed animals must be epigenetic. Among many others, 5-azacytidine, genistein, biochanin A, vinclozolin, and zinc, all of which can alter DNA methylation, were shown to have an effect on body length (Table 1) [34, 56]. This growth effect, however, was transient as it was only seen in 7-day-old animals but not adults [34]. Additionally, zinc exposure significantly reduced body length of 6-day-old animals in the untreated F1 generation [56]. This finding might be an indication of a transgenerationally heritable effect but as it did not persist to the F2 generation, it is more likely the result of embryonic exposure (Table 1).

## 6. Epigenetic Regulation of Fertility

Fertility was also shown to be affected by chemical treatment. While vinclozolin exposure had no significant effect, 5-azacytidine, 5-aza-2′-deoxycytidine, genistein, biochanin A, and cadmium all reduced reproduction in surviving females, in comparison to nonexposed females (Table 1) [34, 57]. Zinc exposure was found to have complex effects; exposure decreased reproductive success in the F0, but not in the subsequent F1 and F2 generations when these were raised in control medium (Table 1) [36]. When animals were continuously exposed to zinc, reproduction was reduced in the F0 and F1 but not the F2 [36]. These results were interpreted as an acclimation effect [36], which would be interesting; however, this conclusion would be strengthened by results from a larger number of reproducing females and corroborating molecular data.

 The effects of chemical exposure occurred in genetically identical individuals and in some cases were heritable between generations, suggesting that the phenotypic variability is epigenetic. This possibility is reinforced by the fact that some of these chemicals have been shown to alter DNA methylation [34].

## 7. Epigenetic Mechanisms—DNA Methylation

The role of epigenetic mechanisms such as DNA methylation, histone modification, and RNAi in normal *Daphnia* development and the epigenetic adaptations described above is still in its infancy. Vandegehuchte et al. [57] were the first to determine that *D. magna* is capable of methylating DNA. They found genes homologous to the three main human DNA methyltransferases and confirmed that DNA methylation occurred. Through the use of ultraperformance liquid chromatography (UPLC) and microarrays, Vandegehuchte et al. [34] examined the DNA methylation and transcription levels, respectively, in *D. magna* exposed to various chemicals. They measured direct effects on methylation in the exposed generation as well as the effects in subsequent generations (Table 1). Global or localized DNA methylation levels were found to be affected by 5-azacytidine, vinclozolin, genistein, and zinc but were not affected by 5-aza-2′-deoxycytidine, biochanin A, and cadmium [34, 57].

5-azacytidine is known to hinder DNA methylation in humans by inhibiting DNA methyltransferases and, consistent with this, *D. magna *treated with 5-azacytidine showed a decrease in global DNA methylation [34, 58]. Interestingly, the untreated offspring of 5-azacytidine exposed daphnid mothers also showed decreased methylation when compared to nonexposed daphnids of the same generation (Table 1). Vandegehuchte et al. [34] interpreted this as a transgenerational effect; however, the F1 were exposed to the toxicant as embryos, a time shown to be sensitive to epigenetic perturbations in many animals [20, 59, 60] including *Daphnia* [51] so these results are more likely due to exposure of the F1 as embryos rather than a true transgenerational effect. Conclusive evidence for a transgenerational effect would be the persistence of the effect into nonexposed generations beyond the F2, a result not observed in this series of experiments. The sensitivity of this experiment and confirmation of any transgenerational effects would be enhanced by examination of gene-specific epigenetic alterations as opposed to global DNA methylation levels, and monitoring changes persisting to the F3 and subsequent generations.

In comparison to nonexposed daphnids, when the F0 was exposed to zinc, there was decreased methylation of the F0 and F1 generations followed by a significant increase in the F2 generation (Table 1) [37]. Vandegehuchte et al. [37] attributed the increase in the third generation to acclimation. While possible, this explanation cannot be confirmed until the study is repeated with a larger sample size. Additionally, as age affects DNA methylation levels in *Daphnia* [37] the age of the daphnids would have to be tightly controlled. Treatment with vinclozolin showed a significant decrease in DNA methylation in *D. magna *in the F0 and F1 exposed generations; however, the F2 showed a nonsignificant increase in overall methylation levels [34]. This implies that while the fungicide vinclozolin does alter DNA methylation, evidence for a transgenerational effect is still lacking. Unusual results were seen with genistein treatment. In mammals, genistein causes global DNA hypomethylation [61] but in *D. magna* it yielded hypermethylated DNA [34]. This confounding result could be attributed to differences in genomic organization between mammals and daphnids, the possibility exists that the sequences that are hypermethylated in the much larger daphnid genome do not exist in humans.

The microarray platform used for these studies was originally designed for investigation of developmentally regulated genes and allowed monitoring of only a subset of those genes, so it was not ideal for global transcription assessment [36]. Until the *D. magna* genome is fully sequenced and a more complete microarray can be employed, it would be preferable to monitor specific genes or to use a species with a fully sequenced genome, such as *D. pulex*. Additionally, bisulfite sequencing, methylated DNA immunoprecipitation (meDip), or DNA methylation sensitive restriction enzyme digests, which allow monitoring of the methylation status of individual genes would be more biologically informative. Candidate genes include those that are involved in reproduction and growth since brood size and body length is affected by toxicant exposure in *D. magna *[34, 36, 56, 57], sex determination [47], as well as those involved in helmet and neckteeth formation [16, 51].

## 8. Conclusion

*Daphnia* have the potential to be invaluable animals for epigenetic study. They are already well-studied in the context of their important ecological and evolutionary roles, as well as being readily available and inexpensive to maintain. The ability of *Daphnia* to produce clones parthenogenetically allows for the elimination of genetic variability, a valuable resource in the study of epigenetics. Obvious phenotypic assay systems such as sexual reproduction, helmets, neckteeth, growth, and fertility allow correlations to be made between such phenotypic responses and the epigenetic changes that accompany them (Table 1). Further, potential transgenerational effects in the production of polyphenisms, intersex individuals, and other epigenetically determined states remain to be explored [44, 45]. There are also many classical and molecular tools available for use in studying epigenetics in *Daphnia*.

 The next steps in establishing *Daphnia* as an epigenetic model organism will be to determine the genetic and epigenetic mechanisms responsible for the establishment and maintenance of phenotypic responses to the environment such as sexual reproduction, helmets, and neckteeth. It is also essential to extend the research on epigenetic mechanisms to include histone modifications, RNAi, and further define the baseline levels and changes in DNA methylation in response to environmental stimuli throughout development. Documenting the epigenetic differences between sexual and asexual *Daphnia* and stressed and unstressed individuals would prove a fruitful area of research with important implications for evolutionary and developmental biology.

## Figures and Tables

**Table 1:** Epigenetic assay systems.

Assay system	Species	F0 treatment	F0 effects	F1	F2	Reference
Helmets	*D. cucullata*	Kairomones	n.d.	Increase	Increase	[16]
	*D. cucullata*	Kairomones	Increase	n.d.	n.d.	[29]
	*D. lumholtzi*	Kairomones	Increase	n.d.	n.d.	[29]
	*D. ambigua*	Kairomones	Increase	n.d.	n.d.	[29]

Neckteeth	*D. pulex*	Kairomones	Increase	n.d.	n.d.	[29]

Growth	*D. magna*	5-azacytidine	Decrease (day 7 only)	Decrease	Decrease (day 7 only)	[34]
		Genistein	Decrease	n.s.	n.s.	[34]
		Vinclozolin	Decrease	n.s.	n.s.	[34]
		Zinc	Decrease (day 6 only)	Decrease (day 6 only)	n.s.	[35]

Reproduction	*D. magna*	5-azacytidine	Decrease	Decrease	n.s.	[34]
		Genistein	Decrease	n.s.	n.s.	[34]
		Vinclozolin	n.s.	n.s.	n.s.	[34]
		Zinc	Decrease	n.s.	n.s.	[36]

Global DNA methylation	*D. magna*	Zinc	n.s.	Decrease	Increase	[37]
		5-azacytidine	Decrease	Decrease	Decrease	[34]
		Genistein	n.s.	n.s.	n.s.	[34]
		Vinclozolin	Decrease	n.s.	Decrease	[34]

Data summarized here is for a treated F0 generation with subsequent generations untreated. n.s. denotes nonsignificant results. n.d. denotes that those trials were not done.
